# Comprehensive Flow-Cytometric Quality Assessment of Ram Sperm Intended for Gene Banking Using Standard and Novel Fertility Biomarkers

**DOI:** 10.3390/ijms23115920

**Published:** 2022-05-25

**Authors:** Jaromír Vašíček, Andrej Baláži, Andrea Svoradová, Jakub Vozaf, Linda Dujíčková, Alexander V. Makarevich, Miroslav Bauer, Peter Chrenek

**Affiliations:** 1 Institute of Farm Animal Genetics and Reproduction, NPPC, Research Institute for Animal Production Nitra, Hlohovecká 2, 951 41 Lužianky, Slovakia; andrej.balazi@nppc.sk (A.B.); andrea.svoradova@nppc.sk (A.S.); linda.dujickova@nppc.sk (L.D.); alexander.makarevic@nppc.sk (A.V.M.); miroslav.bauer@nppc.sk (M.B.); 2 Institute of Biotechnology, Faculty of Biotechnology and Food Science, Slovak University of Agriculture in Nitra, Tr. A. Hlinku 2, 949 76 Nitra, Slovakia; xvozaf@uniag.sk; 3 Department of Morphology, Physiology and Animal Genetics, Faculty of Agri Sciences, Mendel University in Brno, Zemědělská 1/1665, 613 00 Brno, Czech Republic; 4 Department of Botany and Genetics, Faculty of Natural Sciences, Constantine the Philosopher University in Nitra, Nábrežie Mládeže 91, 949 74 Nitra, Slovakia

**Keywords:** ram, native breeds, semen, flow cytometry, biomarkers, ubiquitin, MKRN1, SPTRX-3, PAWP, H3K4me2

## Abstract

Flow cytometry becomes a common method for analysis of spermatozoa quality. Standard sperm characteristics such as viability, acrosome and chromatin integrity, oxidative damage (ROS) etc. can be easily assess in any animal semen samples. Moreover, several fertility-related markers were observed in humans and some other mammals. However, these fertility biomarkers have not been previously studied in ram. The aim of this study was to optimize the flow-cytometric analysis of these standard and novel markers in ram semen. Ram semen samples from Slovak native sheep breeds were analyzed using CASA system for motility and concentration and were subsequently stained with several fluorescent dyes or specific antibodies to evaluate sperm viability (SYBR-14), apoptosis (Annexin V, YO-PRO-1, FLICA, Caspases 3/7), acrosome status (PNA, LCA, GAPDHS), capacitation (merocyanine 540, FLUO-4 AM), mitochondrial activity (MitoTracker Green, rhodamine 123, JC-1), ROS (CM-H_2_DCFDA, DHE, MitoSOX Red, BODIPY), chromatin (acridine orange), leukocyte content, ubiquitination and aggresome formation, and overexpression of negative biomarkers (MKRN1, SPTRX-3, PAWP, H3K4me2). Analyzed semen samples were divided into two groups according to viability as indicators of semen quality: Group 1 (viability over 60%) and Group 2 (viability under 60%). Significant (*p* < 0.05) differences were found between these groups in sperm motility and concentration, apoptosis, acrosome integrity (only PNA), mitochondrial activity, ROS production (except for DHE), leukocyte and aggresome content, and high PAWP expression. In conclusion, several standard and novel fluorescent probes have been confirmed to be suitable for multiplex ram semen analysis by flow cytometry as well as several antibodies have been validated for the specific detection of ubiquitin, PAWP and H3K4me2 in ram spermatozoa.

## 1. Introduction

Cryopreservation of the animal semen belongs to the widely used tools for the preservation either agriculturally important or endangered animals. However, semen samples (spermatozoa) entering the cryopreservation process have to be of a very good quality in order to achieve high cryosurvival rates after their thawing. This requirement is much more important in the case of animal species such as sheep, whose spermatozoa are highly sensitive to oxidative damage [1] or any cold shock [2]. From the basic indicators of semen quality, spermatozoa motility and concentration are assumed to be the most important [3]. Nevertheless, when sperm conservation of valuable national breeds is planned, it is not wise to rely only on motility characteristics obtained using basic microscopic assessment [4], which cannot reveal hidden cell defects possibly resulting in worse or poor quality of thawed samples. On the other hand, flow cytometry offers a much deeper analysis of spermatozoa quality, and thus, this method has become a standard laboratory technique for such analyses [5,6].

Standard sperm flow-cytometric analyses involved assessment of different spermatozoa characteristics, which more or less affect the overall semen quality. The viability of spermatozoa basing on their plasma membrane integrity is commonly analyzed either directly using SYBR-14 dye, staining of live metabolically active cells, or in combination with dead cell dyes, entering to cells via disrupted membrane, such as propidium iodide (PI) [7] or 7-amino-actinomycin-D (7-AAD). 7-AAD, in comparison to PI, can avoid the interference between green and orange fluorescence [8]. Moreover, dead cell dyes are often used alone, without SYBR-14, to check the membrane integrity. Recently, a novel far-red fluorescent viability dye, DRAQ7, that stains nuclei in dead or membrane-compromised cells has been validated for even long-term monitoring of cell health using microcopy and flow cytometry [9]. This nontoxic dye is suitable for multiparametric analysis by flow cytometry because it can be easily combined with common fluorochromes as FITC, PE, etc. In addition to viability analysis, apoptosis-like changes in spermatozoa should be analyzed because such spermatozoa can be hidden within the live cell population. The most often evaluated changes are translocation of phosphatidylserine (PS) to the outer leaflet of plasma membrane detected using Annexin V [10]; increased membrane permeability revealed by nuclear dye YO-PRO-1 iodide [11]; and activation of caspases in general or even specific caspases detected using different reagents such as FLICA (fluorescent inhibitor of caspases) [12]. When sperm membrane integrity is corrupted, an acrosomal membrane is exposed indicating the damaged or reacted acrosome. To detect this issue, most often lectins, mainly *Pisum sativum* (pea) agglutinin (PSA) and *Arachis hypogaea* (peanut) agglutinin (PNA) [13] or rarely also *Lens culinaris* agglutinin (LCA) [14], are used. Recently, the binding of a Hs-8 monoclonal antibody to an intra-acrosomal protein GAPDHS (sperm-specific glyceraldehyde phosphate dehydrogenase) was reported to evaluate sperm acrosome [15,16]. Capacitation is essential for fertilization process and comes before the acrosome reaction. Changes reporting capacitation on the membrane level can be assessed using merocyanine 540 (M540) [17,18]. However, as alternative to M540, Ca^2+^ fluorescent probes FLUO-3 AM [19] or FLUO-4 AM [20] for detecting intracellular Ca^2+^ in spermatozoa, which better indicate capacitation process, have been used in the last decade.

Besides high viability, increased mitochondrial activity is another important indicator of good spermatozoa quality. Activity of mitochondria can be measured through membrane mitochondrial potential (MMP) with the use of several fluorescent dyes like rhodamine 123 [21], JC-1 or MitoTracker probes [22]. Oxidative stress or damage triggered by reactive oxygen species (ROS) may have unfavorable impact on spermatozoa fertilizing ability. There are several probes, which accumulate in cells and become fluorescent after oxidation and, thus, may be used for the detection of ROS in spermatozoa. Some of them are unspecific, such as 2′,7′-dichlorodihydrofluorescein diacetate (H_2_DCFDA) and its derivate CM-H_2_DCFDA [23], whereas others detect specific oxidant species, e.g., dihydroethidium (DHE) [24] or MitoSOX Red [25] reacting with the superoxide anion (O_2_^−^), or BODIPY probes detecting lipid peroxidation of membranes [26]. Increased ROS production in men ejaculates has been shown to be related to the presence of leukocytes [27]. However, leukocytes presented in semen can be also a sign of hidden inflammatory process. Other than a microscopic evaluation of leukocytes in semen [28], an antibody-based detection of leukocytes using flow cytometry can also be performed [29]. A sperm DNA damage caused by any of possible factors is routinely assessed by flow cytometry using acridine orange (AO) dye for more than two decades [30] or alternatively by TUNEL technique [31].

All above mentioned sperm characteristics and physiological changes are standardly assessed in human or animal semen samples via flow cytometry. However, novel biomarkers have been reported recently, which may be related with the spermatozoa fertility and can be evaluated using flow cytometry. It has been noticed that abnormal ubiquitinated spermatozoa presented in the ejaculate indicate a poor semen quality [32]. In general, ubiquitination is a mechanism of apoptosis, in which a small ubiquitin protein is inserted into the sperm plasma membrane. Ubiquitinated spermatozoa are usually removed in the testis by proteosome [33] or in the epididymis by phagocytosis [34]. Despite those mechanisms, such spermatozoa can emerge also in semen samples, where they can be detected by staining with specific antibodies [32]. An alternative to ubiquitin antibodies, aggregates of ubiquitinated proteins in spermatozoa can be labeled using specific Proteostat aggresome detection kit [35].

In addition to ubiquitin, expression of some other markers in spermatozoa have been reported to negatively correlate with fertility, i.e., MKRN1, SPTRX-3, PAWP and H3K4me2. MKRN1, Makorin ring finger protein-1, is conserved in mammals and highly expressed in adult testis [36]. Defective spermatozoa with accumulated MKRN1 were noticed in human and bull semen samples associated with male infertility (Sutovsky; unpublished data). SPTRX-3, spermatid specific thioredoxin-3 (known also as TXNDC8), is a member of thioredoxin family specific for male/testis germline. It has been observed to accumulate in the superfluous midpiece cytoplasm and nuclear vacuoles of defective human spermatozoa, or in the round testicular spermatids of other mammals. An increased content of SPTRX-3 positive spermatozoa was found in infertile males [37,38]. PAWP, post-acrosomal sheath WW-domain binding protein (known also as WBP2NL), is located in the post-acrosomal region of heads in normal spermatozoa and plays an important role during fertilization in the oocyte activation. Medium levels of PAWP were detected in normal spermatozoa. On the other hand, low or high levels of PAWP were associated with poor fertility and abnormal sperm morphology [38]. H3K4me2, histone H3 dimethylated on lysine K4, is an epigenetic marker, which appears to be associated with decreased sperm quality. Dimethylation of H3K4 belongs to the posttranslational modifications of histones in the sperm heads during spermatogenesis. However, the excess of H3K4me2 modification may indicate defects in chromatin integrity [39].

After all, the main goal of this study was to establish a multiparametric flow cytometry panel of standard and novel markers indicating good or poor quality of ram spermatozoa, which can be potentially used to evaluate semen samples from valuable breeding males prior to their further processing such as cryopreservation. The main advantage of such deep evaluation of sperm quality is to reveal hidden physiological alterations, which may have after all negative impact on the cryosurvival rate of individual sperm samples as well as on the reproductive outcomes, such as artificial insemination, in vitro fertilization etc. Moreover, this study can help other researchers to orient in the variety of available flow-cytometric sperm quality probes and choose the proper ones for their own research.

## 2. Results

### 2.1. Sperm Viability, Apoptosis and CASA Parameters

**Figure 1:**
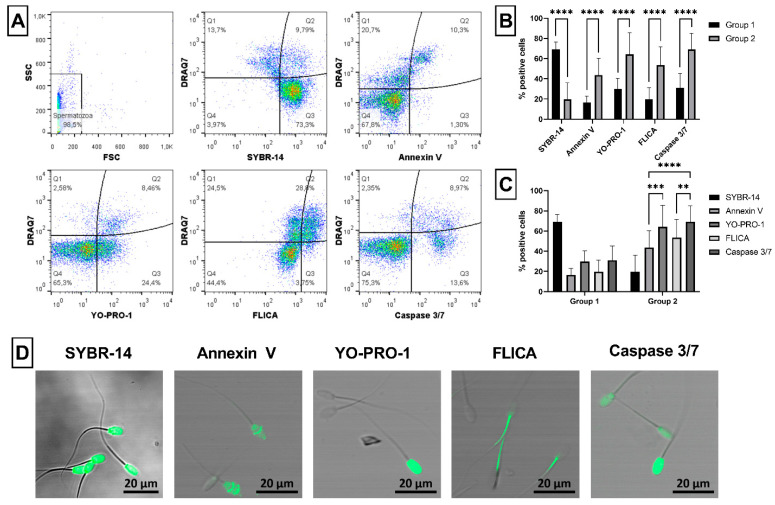
Evaluation of ram sperm viability and apoptosis. Illustrative flow-cytometric dot plots showing evaluation strategy of analyzed fluorescent probes. Firstly, spermatozoa were gated using FSC/SSC dot plot. Then, dot plots showing dead spermatozoa in far-red FL4 channel (DRAQ7^+^) and live (SYBR-14^+^) or apoptotic (Annexin V^+^, YO-PRO-1^+^, FLICA^+^ and Caspase 3/7^+^) spermatozoa in green FL1 channel were created and divided into four quadrants (Q) according to fluorescent signals (**A**). Graph showing comparison of live (Q3: SYBR-14^+^/DRAQ7^−^) and apoptotic spermatozoa detected using Annexin V (Q2: AnV^+^/DRAQ7^+^ and Q3: AnV^+^/DRAQ7^−^), YO-PRO-1 (Q2: YO-PRO-1^+^/DRAQ7^+^ and Q3: YO-PRO-1^+^/DRAQ7^−^), FLICA (Q2: FLICA^+^/DRAQ7^+^ and Q3: FLICA^+^/DRAQ7^−^), and Caspase 3/7 (Q2: Caspase 3/7^+^/DRAQ7^+^ and Q3: Caspase 3/7^+^/DRAQ7^−^) in Group 1 and Group 2 (**B**). Graph comparing proportion of apoptotic spermatozoa detected using four different fluorescent probes (Annexin V, YO-PRO-1, FLICA and Caspase 3/7) within each group (**C**). Illustrative images from confocal microscopy (Zeiss LSM 700) proving the specific staining of ram spermatozoa with live and apoptotic fluorescent probes (magnification at 200×, scale bar = 20 µm). SYBR-14 specifically labeled DNA in the nucleus of spermatozoa with intact membrane (green), whereas YO-PRO-1 stained the nucleus of apoptotic cells (green). Annexin V labeled deteriorated membranes in different parts of sperm heads (green). FLICA reagent specifically stained active poly caspases in sperm tails (green), while Caspase 3/7 labeled active caspases 3/7 mainly in sperm heads (green) (**D**). Group 1—semen samples with sperm viability over 60%, Group 2—semen samples with sperm viability under 60%. The data are expressed as the means ± SD; difference is statistically significant at ** *p* < 0.01, *** *p* < 0.001 and **** *p* < 0.0001.

Ram semen samples analyzed in this study were divided into two groups according to the sperm viability assessed using combination of SYBR-14 and DRAQ7 dyes (Figure 1A). Samples in Group 1 contained more than 60% of viable (SYBR-14^+^/DRAQ7^−^) spermatozoa (in average about 70%), while samples in Group 2 contained less than 60% of viable spermatozoa (in average about 20%). The difference in the viability between these groups was significant (*p* < 0.0001; 69.3 ± 7.2% vs. 19.7 ± 16.2%, respectively; Figure 1B). Moreover, significantly (*p* < 0.0001) increased expression of all apoptotic markers (Annexin V, YO-PRO-1, FLICA and Caspase 3/7) was detected in Group 2 compared to Group 1. In addition, significant differences were found in Group 2 between proportion of Annexin V positive (AnV^+^) and YO-PRO-1^+^ spermatozoa (*p* < 0.001) or between spermatozoa positive for FLICA and Caspase 3/7 (*p* < 0.01). Likewise, a significantly (*p* < 0.0001) higher presence of apoptotic spermatozoa was detected using Caspase 3/7 in comparison to Annexin V in Group 2 (Figure 1C). The specific staining with viable (SYBR-14) or apoptotic (Annexin V, YO-PRO-1, FLICA and Caspase 3/7) probes was confirmed by confocal microscopy (Figure 1D).

**Figure 2:**
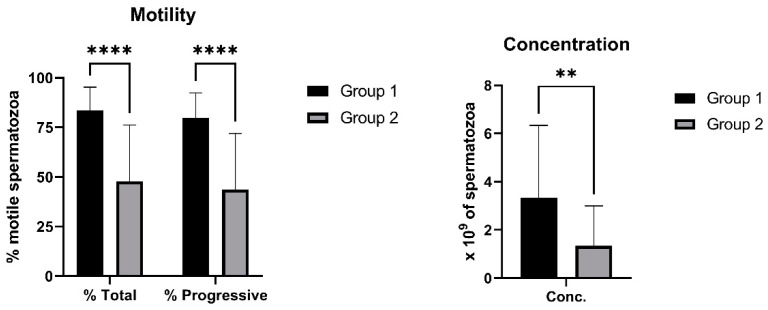
CASA parameters of analyzed ram semen sample according to the sperm viability. Group 1—semen samples with sperm viability over 60%, Group 2—semen samples with sperm viability under 60%, % Total—percentage of totally motile spermatozoa, % Progressive—percentage of progressively motile spermatozoa, Conc.—concentration of spermatozoa. The data are expressed as the means ± SD; difference is statistically significant at ** *p* < 0.01 and **** *p* < 0.0001.

The motility parameters (total and progressive motility) as well as the concentration of spermatozoa were significantly higher (*p* < 0.0001, *p* < 0.0001 and *p* < 0.01, respectively) in Group 1 compared to Group 2 (Figure 2). Furthermore, the spermatozoa motility did not always correlate with their viability (data not shown). Some sperm samples had worse viability (under 60% and/or much less), despite their high motility values resulting in higher SD values in Group 2 (Figure 2).

### 2.2. Sperm Acrosome Integrity and Capacitation Status

**Figure 3:**
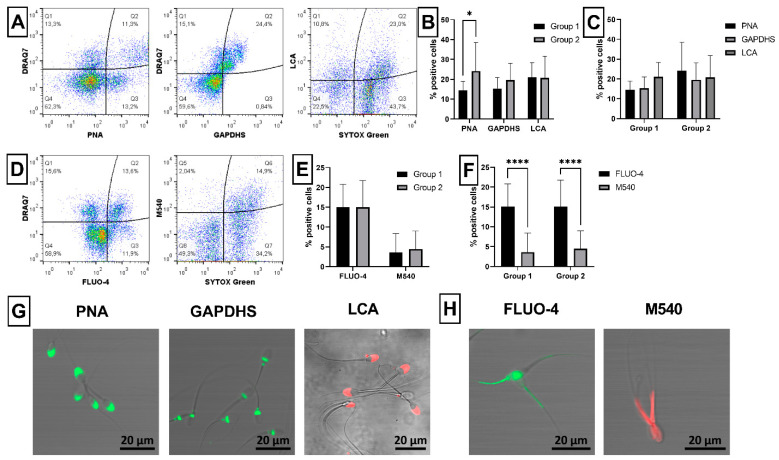
Evaluation of ram sperm acrosome integrity and capacitation status. Illustrative flow-cytometric dot plots showing evaluation strategy of analyzed fluorescent probes. Spermatozoa gated using FSC/SSC dot plot showing dead spermatozoa in far-red FL4 channel (DRAQ7^+^) or green FL1 channel (SYTOX Green) and acrosome-damaged spermatozoa in green FL1 channel (PNA and GAPDHS) or red FL3 channel (LCA) were divided into four quadrants (Q) according to fluorescent signals (**A**). Graph showing comparison of acrosome-damaged spermatozoa detected using PNA (Q2: PNA^+^/DRAQ7^+^ and Q3: PNA^+^/DRAQ7^−^), GAPDHS (Q2: GAPDHS^+^/DRAQ7^+^ and Q3: GAPDHS^+^/DRAQ7^−^), and LCA (Q1: LCA^+^/SYTOX Green^−^ and Q2: LCA^+^/SYTOX Green^+^) in Group 1 and Group 2 (**B**). Graph comparing proportion of acrosome-damaged spermatozoa detected using three different fluorescent probes (PNA, GAPDHS and LCA) within each group (**C**). Illustrative flow-cytometric dot plots showing evaluation strategy of analyzed fluorescent probes. Spermatozoa gated using FSC/SSC dot plot showing dead spermatozoa in far-red FL4 channel (DRAQ7^+^) or FL1 channel (SYTOX Green) and capacitated spermatozoa in green FL1 channel (FLUO-4) or red FL3 channel (M540) were divided into four quadrants (Q) according to fluorescent signals (**D**). Graph showing comparison of capacitated spermatozoa detected using FLUO-4 (Q2: FLUO-4^+^/DRAQ7^+^ and Q3: FLUO-4^+^/DRAQ7^−^) and M540 (Q5: M540^+^/SYTOX Green^−^ and Q2: M540^+^/SYTOX Green^+^) in Group 1 and Group 2 (**E**). Graph comparing proportion of capacitated spermatozoa detected using two different fluorescent probes (FLUO-4 and M540) within each group (**F**). Illustrative images from confocal microscopy (Zeiss LSM 700; magnification at 200×, scale bar = 20 µm) showing the staining pattern of ram spermatozoa with acrosomal fluorescent probes PNA (green) and LCA (red) specifically stained sperm acrosome, while GAPDHS antibody (green) labeled post-acrosomal region of sperm heads (**G**), and probes for sperm capacitation FLUO-4 (green) and M540 (red) labeled sperm heads and tails (**H**). Group 1—semen samples with sperm viability over 60%, Group 2—semen samples with sperm viability under 60%. The data are expressed as the means ± SD; difference is statistically significant at * *p* < 0.05 and **** *p* < 0.0001.

Acrosomal status of ram spermatozoa was assessed using three different probes (PNA, GAPDHS and LCA; Figure 3A). Significantly (*p* < 0.05) increased proportion of spermatozoa with damaged acrosome was detected only using PNA in Group 2 compared to Group 1 (Figure 3B). However, no significant differences were found among the proportion of PNA^+^, GAPDHS^+^ or LCA^+^ spermatozoa within each group (Figure 3C). Confocal microscopy confirmed specific staining of sperm acrosome by PNA and LCA, whereas GAPDHS antibody bound nonspecifically to the post-acrosomal part of sperm heads (Figure 3G). The capacitation status of ram spermatozoa was evaluated using two different probes (FLUO-4 and M540; Figure 3D). No significant differences between groups were found in the proportion of capacitated spermatozoa detected either by FLUO-4 or M540 (Figure 3E). However, a significant (*p* < 0.0001) difference was found between proportion of FLUO-4^+^ and M540^+^ spermatozoa in each group (Figure 3F). Specific staining of both probes for sperm capacitation was proved by confocal microscopy (Figure 3H).

### 2.3. Sperm Mitochondrial Activity

**Figure 4:**
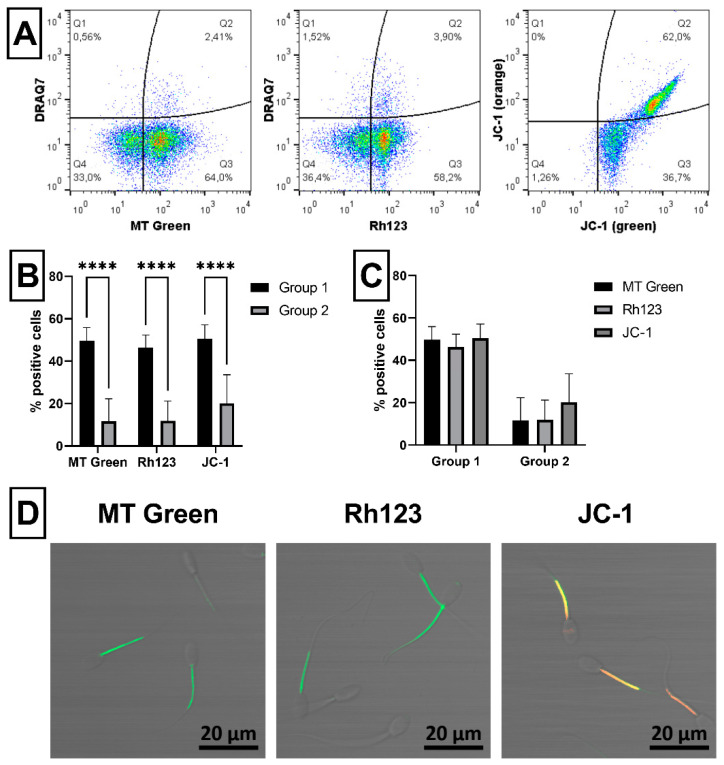
Evaluation of ram sperm mitochondrial activity. Illustrative flow-cytometric dot plots showing evaluation strategy of analyzed fluorescent probes. Spermatozoa gated using FSC/SSC dot plot showing dead spermatozoa in far-red FL4 channel (DRAQ7^+^) and spermatozoa with high MMP in green FL1 channel (MT Green and Rh123) or orange FL2 channel (JC-1) were divided into four quadrants (Q) according to fluorescent signals (**A**). Graph showing comparison of spermatozoa with high MMP detected using MT Green (Q3: MT Green^+^/DRAQ7^−^), Rh123 (Q3: Rh123^+^/DRAQ7^−^), and JC-1 (Q2: JC-1 orange aggregates) in Group 1 and Group 2 (**B**). Graph comparing proportion of spermatozoa with high MMP detected using three different fluorescent probes (MT Green, Rh123 and JC-1) within each group (**C**). Illustrative images from confocal microscopy (Zeiss LSM 700) proving the specific staining of ram spermatozoa with active mitochondria (magnification at 200×, scale bar = 20 µm). MT Green and Rh123 (green), and JC-1 (orange) specifically labeled the mitochondrial region of spermatozoa tails (**D**). Group 1—semen samples with sperm viability over 60%, Group 2—semen samples with sperm viability under 60%. The data are expressed as the means ± SD; difference is statistically significant at **** *p* < 0.0001.

The activity of ram sperm mitochondria was assessed via mitochondrial membrane potential (MMP) using three different fluorescent probes: MitoTracker Green (MT Green), Rhodamine 123 (Rh123) and JC-1 (Figure 4A). Significantly (*p* < 0.0001) increased presence of spermatozoa with high MMP was detected using all three probes in Group 1 compared to Group 2 (Figure 4B). On the other hand, no differences were observed among the proportion of MT Green^+^, Rh123^+^ or JC-1^+^ spermatozoa within each group (Figure 4C). Specific staining of the mitochondrial region of spermatozoa tails by all three probes was confirmed by confocal microscopy (Figure 4D).

### 2.4. Sperm ROS Generation

**Figure 5:**
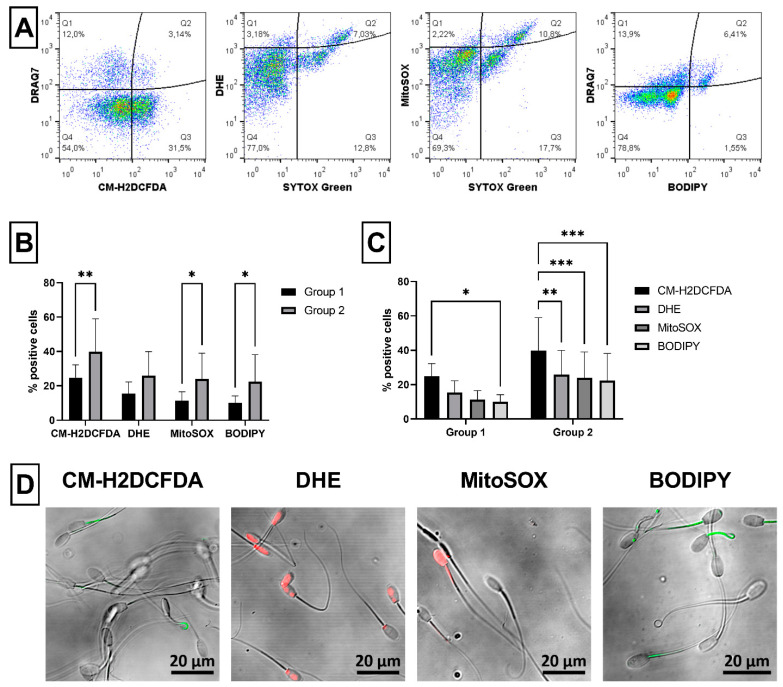
Evaluation of ROS generation in ram semen samples. Illustrative flow-cytometric dot plots showing evaluation strategy of analyzed fluorescent probes. Spermatozoa gated using FSC/SSC dot plot showing dead spermatozoa in far-red FL4 channel (DRAQ7^+^) or green FL1 channel (SYTOX Green) and ROS positive spermatozoa in green FL1 channel (CM-H_2_DCFDA and BODIPY) or red FL3 channel (DHE and MitoSOX) were divided into four quadrants (Q) according to fluorescent signals (**A**). Graph showing comparison of ROS positive spermatozoa detected using CM-H_2_DCFDA (Q2: CM-H_2_DCFDA^+^/DRAQ7^+^ and Q3: CM-H_2_DCFDA^+^/DRAQ7^−^), DHE (Q1: DHE^+^/SYTOX Green^−^ and Q2: DHE^+^/SYTOX Green^+^), MitoSOX (Q1: MitoSOX^+^/SYTOX Green^−^ and Q2: MitoSOX^+^/SYTOX Green^+^) and BODIPY (Q2: BODIPY^+^/DRAQ7^+^ and Q3: BODIPY^+^/DRAQ7^−^) in Group 1 and Group 2 (**B**). Graph comparing proportion of ROS positive spermatozoa detected using four different fluorescent probes (CM-H_2_DCFDA, DHE, MitoSOX and BODIPY) within each group (**C**). Illustrative images from confocal microscopy (Zeiss LSM 700; magnification at 200×, scale bar = 20 µm) showing the staining pattern of ram ROS positive spermatozoa: CM-H_2_DCFDA (green), DHE and MitoSOX (red), and BODIPY (green) stained different parts (heads and tails) of spermatozoa with ROS (**D**). Group 1—semen samples with sperm viability over 60%, Group 2—semen samples with sperm viability under 60%, DHE—dihydroethidium. The data are expressed as the means ± SD; difference is statistically significant at * *p* < 0.05, ** *p* < 0.01 and *** *p* < 0.001.

Production of ROS in ram semen samples was measured using four different fluorescent probes: CM-H_2_DCFDA, DHE, MitoSOX™ Red (MitoSOX) and BODIPY™ 581/591 C11 (BODIPY) (Figure 5A). Significantly increased ROS generation was found in Group 2 compared to Group 1, when detected using CM-H_2_DCFDA (*p* < 0.01), MitoSOX (*p* < 0.05) and BODIPY (*p* < 0.05), but not with DHE probe (Figure 5B). Proportion of ROS positive spermatozoa stained with CM-H_2_DCFDA was significantly (*p* < 0.05) higher than those stained with BODIPY probe in Group 1. Moreover, proportion of CM-H_2_DCFDA^+^ spermatozoa was significantly higher than those labeled with DHE (*p* < 0.01), MitoSOX (*p* < 0.001) or BODIPY (*p* < 0.001) in Group 2 (Figure 5C). The specific staining of ROS positive spermatozoa was confirmed using confocal microscopy (Figure 5D).

### 2.5. Sperm Chromatin Status and Leukocyte Detection

**Figure 6:**
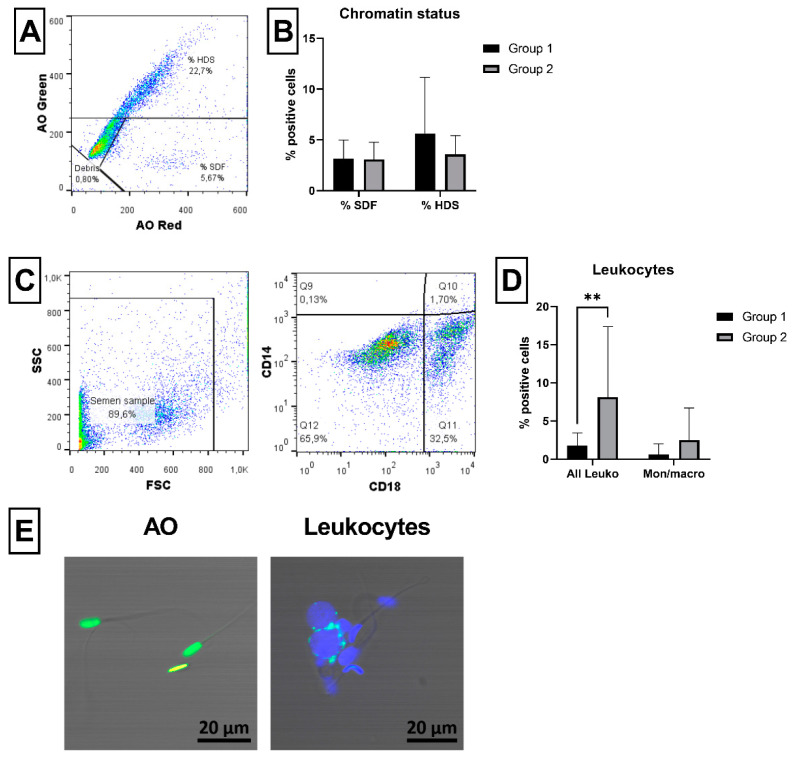
Evaluation of ram sperm chromatin damage and leukocytes occurrence. Illustrative flow-cytometric dot plots showing evaluation strategy of analyzed AO probe. Spermatozoa were evaluated in a linear scale of green FL1 channel and red FL3 channel, where spermatozoa bearing loose chromatin shifted fluorescence to red (% SDF) and immature spermatozoa showed high green fluorescence (% HDS) (**A**). Graph showing comparison of spermatozoa with fragmented DNA (% SDF) and spermatozoa with high DNA stainability (% HDS) in Group 1 and Group 2 (**B**). Illustrative flow-cytometric dot plots showing evaluation strategy of analyzed fluorescent antibodies. Spermatozoa and somatic cells excluded of dead cells (DRAQ7^+^) were gated using FSC/SSC, showed in green FL1 channel (CD18) and orange FL2 channel (CD14) and divided into four quadrants (Q) according to fluorescent signals (**C**). Graph showing comparison of all leukocytes (Q10: CD18^+^/CD14^+^ and Q11: CD18^+^/CD14^−^) and monocytes/macrophages alone (Q10: CD18^+^/CD14^+^) detected in Group 1 and Group 2 (**D**). Illustrative images from confocal microscopy (Zeiss LSM 700; magnification at 200×, scale bar = 20 µm) proving the specific staining of ram spermatozoa with damaged chromatin (AO: orange nucleus) and specific membrane staining of leukocytes (CD18, green) and nucleus (DAPI, blue) (**E**). AO—acridine orange, % SDF—spermatozoa with fragmented DNA, % HDS—high DNA stainability (immature spermatozoa), Group 1—semen samples with sperm viability over 60%, Group 2—semen samples with sperm viability under 60%, Leuko—leukocytes, mon/macro—monocytes/macrophages. The data are expressed as the means ± SD; difference is statistically significant at ** *p* < 0.01.

Damage of ram sperm DNA was evaluated using acridine orange (AO) dye, where the proportion of spermatozoa with fragmented DNA (% SDF) and immature spermatozoa (% HDS) was observed (Figure 6A). No significant differences were found in percent SDF and percent HDS in Group 1 compared to Group 2 (Figure 6B). The presence of leukocytes and monocytes/macrophages in ram semen samples was detected using specific antibodies (CD18 and CD14, respectively; Figure 6C). Significantly (*p* < 0.01) more leukocytes (CD18^+^ cells) were noticed in Group 2 compared to Group 1. However, no difference was noticed in the content of monocytes/macrophages (CD18^+^/CD14^+^ cells) between the groups (Figure 6D). Confocal microscopy confirmed the specific staining of spermatozoa with fragmented DNA and specific membrane staining of leukocytes (Figure 6E).

### 2.6. Sperm Ubiquitination and Formation of Aggresomes

**Figure 7:**
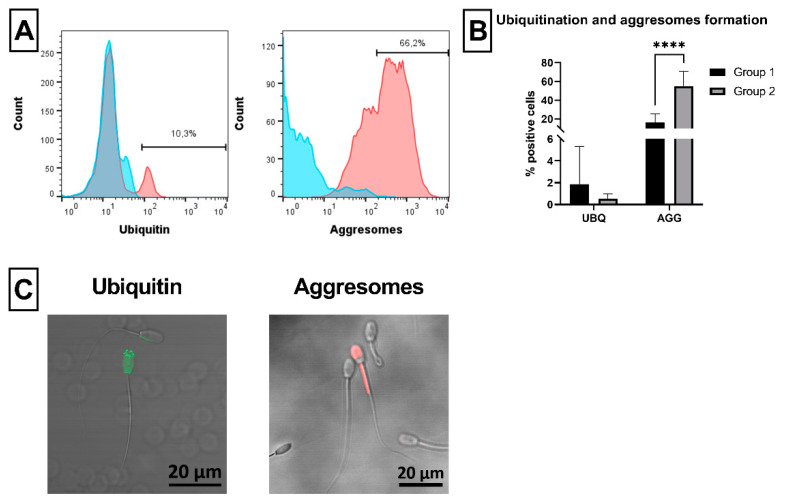
Evaluation of defective spermatozoa with ubiquitinated proteins and aggresomes. Illustrative flow-cytometric histograms showing evaluation strategy of analyzed fluorescent markers. Spermatozoa gated using FSC/SSC dot plot were showed using histogram in green FL1 channel (UBQ) or red FL3 channel (Aggresomes), where a control unstained population (blue) was compared to positively stained population (red) according to fluorescent signals (**A**). Graph showing comparison of defective spermatozoa detected using anti-ubiquitin antibody (UBQ) or aggresomes kit (AGG) in Group 1 and Group 2 (**B**). Illustrative images from confocal microscopy (Zeiss LSM 700; magnification at 200×, scale bar = 20 µm) showing the specific staining of ram ubiquitinated spermatozoa (green) and spermatozoa with aggresomes (red) (**C**). Group 1—semen samples with sperm viability over 60%; Group 2—semen samples with sperm viability under 60%. The data are expressed as the means ± SD; difference is statistically significant at **** *p* < 0.0001.

Defective ram spermatozoa with ubiquitinated proteins were detected using anti-ubiquitin antibody (UBQ). In addition, aggregates of these ubiquitinated proteins (aggresomes) were also observed in this study (Figure 7A). No difference was found in the proportion of UBQ positive spermatozoa between groups. On the contrary, a significant (*p* < 0.0001) increase of spermatozoa with aggresomes was noticed in Group 2 compared to Group 1 (Figure 7B). Positive staining of spermatozoa with ubiquitinated proteins or aggresomes was confirmed by confocal microscopy (Figure 7C).

### 2.7. Novel Sperm Biomarkers Associated with Fertility

**Figure 8:**
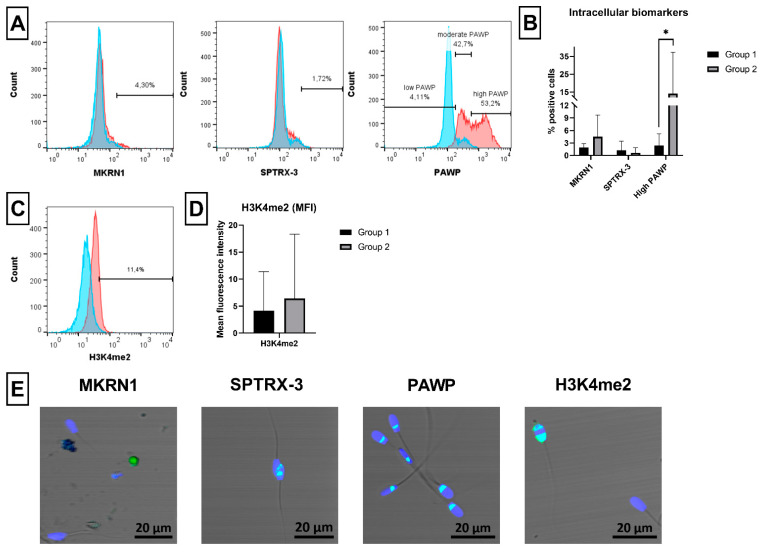
Evaluation of defective spermatozoa with overexpression of MKRN1, SPTRX-3, PAWP and H3K4me2. Illustrative flow-cytometric histograms showing evaluation strategy of analyzed fluorescent markers. Spermatozoa gated using FSC/SSC dot plot were showed using histogram in green FL1 channel (MKRN1, SPTRX-3 and PAWP), where a control unstained population (blue) was compared to positively stained population (red) according to fluorescent signals. Two positive populations (moderate and high) can be distinguished for PAWP according to the fluorescent intensity (**A**). Graph showing comparison of defective spermatozoa positive for MKRN1 and SPTRX-3 or highly positive for PAWP (high PAWP) in Group 1 and Group 2 (**B**). Illustrative flow-cytometric histogram showing analyzed fluorescent marker. Spermatozoa gated using FSC/SSC dot plot were showed using histogram in green FL1 channel (H3K4me2), where a control unstained population is in blue color and positively stained population is in red color (**C**). Graph showing comparison of defective spermatozoa positive for H3K4me2 in Group 1 and Group 2. The final value for mean fluorescence intensity (MFI) of H3K4me2 was obtained after subtracting the MFI of control sample stained only with secondary antibody from the signal (MFI) of the experimental sample (**D**). Illustrative images from confocal microscopy (Zeiss LSM 700; magnification at 200×, scale bar = 20 µm) showing the staining of ram semen samples by different antibodies (green) and DAPI (blue: nucleus) (**E**). Group 1—semen samples with sperm viability over 60%, Group 2—semen samples with sperm viability under 60%. The data are expressed as the means ± SD; difference is statistically significant at * *p* < 0.05.

Defective ram spermatozoa with increased expression of some novel fertility related markers were evaluated using specific antibodies against MKRN1, SPTRX-3, PAWP and H3K4me2 (Figure 8A,C). No differences were found in expression of MKRN1 and SPTRX-3 by spermatozoa between both groups. However, the expression of these markers was very low independently of the studied group (Figure 8A,B). On the other hand, significantly (*p* < 0.05) increased presence of spermatozoa with high PAWP expression was observed in Group 2 compared to Group 1 (Figure 8B). On the contrary, no difference was noticed in the mean fluorescence intensity (MFI) of H3K4me2 between the groups (Figure 8D). The specificity of used antibodies was checked by confocal microscopy. A doubtful fluorescent staining was observed in case of MKRN1 and SPTRX-3, while a specific staining pattern was observed for PAWP and H3K4me2 (Figure 8E).

## 3. Discussion

Present study indicates that motility should not be the decisive indicator of ram spermatozoa quality, mainly if the semen samples are collected from individual breeding males for the purpose of long-term cryopreservation of animal genetic resources. On the contrary, a thorough analysis of different sperm properties, which directly affect the overall semen quality, is much more desirable. For this reason, flow cytometry is the best choice to make such analysis in a relatively short time.

Usually, a minimum of 60–70% motility is required as optimal for boar or bovine spermatozoa to further processing [40,41]. However, analysis of ram semen samples in our study showed that the sperm viability of some samples was poor, even if their motility was relatively high (over 70%, data not shown). This observation may indicate that the motility of ram spermatozoa does not necessary reflect their viability or sperm membrane status. We can assume that even spermatozoa with compromised membrane integrity may have still enough energy for their movement at the time of motility analysis. The other possible reason of the worsened viability of motile spermatozoa in some samples may be their increased sensitivity to environmental conditions and laboratory procedures in comparison to other ram sperm samples. Therefore, a measurement of sperm motility should be always combined with a determination of necrotic and/or apoptotic status, as was reported previously [42,43]. On the other hand, good sperm viability of analyzed samples mostly correlated with high motility of ram spermatozoa. Therefore, we chose the spermatozoa viability as basic indicator of ram semen quality and split the analyzed semen samples into Group 1 with good quality (viability > 60%) and Group 2 with poor semen quality (viability < 60%), which can also be done according to sperm motility. An average spermatozoa motility (total or progressive) in Group 1 was about 80%, which may represent an optimal value for further processing of ram semen or even for cryopreservation. Moreover, the Group 1 involved samples with significantly higher sperm concentration than Group 2 (Figure 2). Similar distribution of semen samples according to sperm viability and motility was performed in the study focused on the quality of thawed stallion spermatozoa [44]. The viability of ram spermatozoa in this study was assessed by the SYBR-14 probe, which is most widely used in combination with PI or 7-AAD [5]. However, to distinguish dead spermatozoa in our samples, a novel far-red dead cell dye DRAQ7 was employed into presented flow-cytometric analysis to fully eliminate the possible spectral overlap of used fluorescent dyes. This dye was successfully used previously for the viability assessment of ram or stallion spermatozoa [44,45]. On the other hand, SYTOX Green, a green dead cell dye, was used in combination with red fluorescent dyes to analyze some sperm-specific attributes in this study. This dye has been also previously reported to be useful for sperm analysis either alone, or in combination with other specific probes, e.g., DRAQ5, Annexin V or DHE [46,47,48].

In this study, semen samples in Group 1 showed increased sperm physiological properties that are positively correlated with sperm quality, such as high MMP, while samples in Group 2 exhibited increased sperm properties negatively correlated with the quality of spermatozoa, such as increased apoptosis, acrosome damage, ROS, leukocytes, etc. In the case of apoptotic-like changes detected in ram spermatozoa, YO-PRO-1 dye seems to be better to label the plasma membrane changes than Annexin V. Similarly, caspase 3/7 probe seems to be more suitable than FLICA to assess caspase activity because higher positivity was observed by using these probes (Figure 1). Furthermore, YO-PRO-1 and Caspase 3/7 probes detected similar proportion of apoptotic ram spermatozoa. YO-PRO-1 has been recently reported to reveal differences in the content of apoptotic cells in frozen-thawed ram spermatozoa under different in vitro capacitation conditions [49] or in rams with high or low fertility [50]. Similarly, Caspase 3/7 probe was useful for apoptosis detection in fresh ram sperm samples exposed to different centrifugal forces [51] or in frozen-thawed samples with various antioxidant supplementation [52]. Moreover, this probe found significantly more positive spermatozoa in cryopreserved stallion semen samples with poor viability and motility [44]. In addition, these stallion samples also displayed significantly decreased mitochondrial activity (high MMP observed by JC-1 dye) in poor samples compared to samples with good viability, similar to our study comparing ram semen samples with good (Group 1) or poor (Group 2) viability (Figure 4). Here, three different mitochondrial probes were tested, MitoTracker Green, Rhodamine 123 and JC-1, with similar results (increased high MMP in Group 1) and no significant differences among them. However, unlike JC-1, MitoTracker dyes can be easily combined with other probes in green or red fluorescent spectrum [5], for example with dead cell marker, such as in our study. Furthermore, MitoTracker dyes are fixable and can be applied to evaluate MMP in ram spermatozoa even after several hours of post-fixation as we reported previously [4]. Several recent studies have been also used MitoTracker probes to observe mitochondrial activity of fresh or frozen-thawed ram spermatozoa [49,50].

The integrity of sperm acrosome is a very important quality feature associated with fertilizing ability of the spermatozoa themselves. Acrosomal status of presented ram sperm samples was assessed by specific binding of lectins—PNA and LCA, or using intra-acrosomal protein antibody (GAPDHS). However, only PNA probe detected significant difference in the content of spermatozoa with damaged acrosome between Group 1 and Group 2 (Figure 3). Moreover, PNA, belonging to the most widely used lectins for spermatozoa assessment [5], can be also fixed, as we demonstrated previously in ram [4]. On the other hand, LCA has been previously used in human [53], boar [54], bovine [14] and mouse [55] spermatozoa, but not in ram sperm samples until now. Interestingly, it was reported that LCA was labeled beside an acrosome of normal spermatozoa and whole sperm heads and tails of defective spermatozoa [14]. Therefore, PNA seems to be more specific for strictly evaluating acrosome integrity of analyzed ram spermatozoa than LCA. For this reason, a GAPDHS antibody (Hs-8), which was generated against human intra-acrosomal protein [16], was also tested in this study. A cross-reactivity of this antibody to boar and mouse spermatozoa has been previously reported [15], as well as the possible binding to ram sperm samples [4]. However, a confocal microscopy analysis, presented in this study, revealed an unspecific binding patter of GAPDHS antibody to post-acrosomal region of the sperm head (Figure 3). Like acrosomal damage, early capacitation of spermatozoa could be also a reason of poor sperm quality and fertilizing ability as well. Although, we did not found differences in the content of early-capacitated spermatozoa between Group 1 and Group 2, significantly more positive spermatozoa were detected using FLUO-4 probe than using M540 dye in both groups (Figure 3). Furthermore, even though M540 is useful for measuring lipid membrane fluidity, it was demonstrated that M540 can also detect membrane degeneration [18]. Therefore, FLUO-4 probe should be more specific for flow-cytometric analysis of sperm capacitation. This probe has been recently used also by other studies to assess capacitated ram spermatozoa [56,57], but not in combination with DRAQ7, as we demonstrated here. The capacitation process itself required an increase in ROS production [58], while an excess of generated ROS is detrimental to spermatozoa [59].

Several types of fluorescent probes can assess unspecific or even specific ROS products in sperm samples. Using CM-H2DCFDA, unspecific ROS probe, significant difference was detected in the proportion of ROS-positive ram spermatozoa between Group 1 and Group 2 (Figure 5). Similarly, other studies used this probe to analyze ROS production in ram semen samples under different conditions of in vitro capacitation [49,50]. Additionally, specific ROS probes, such as MitoSOX and BODIPY but not DHE, also showed increased ROS production in Group 2 compared to Group 1. On the other hand, unspecific CM-H2DCFDA probe labeled higher proportion of ROS positive spermatozoa than the specific probes (Figure 5). Thus, CM-H2DCFDA seems to be a suitable probe for rapid unspecific detection of ROS production in ram semen samples prior their further processing. However, if specific ROS production on mitochondrial or membrane level is preferable, then MitoSOX or BODIPY may be used rather than DHE for ram spermatozoa flow-cytometric assessment. Moreover, DHE dye was reported to produce another red fluorescent product under unspecific oxidation [60,61]. Therefore, a different method than flow cytometry has been suggested for detection of these two different red fluorescent DHE products [62]. At last, alternatively to CM-H2DCFDA, a novel fixable ROS probe, CellROX, may be used to detect unspecific ROS products in fresh and frozen/thawed [51,52] or even post-fixed ram spermatozoa, as we demonstrated previously [4]. Moreover, it was observed that several antioxidants such as melatonin may enhance the antioxidative properties of ram semen [63]. Therefore, flow-cytometric ROS probes tested in this study can be used to effectively assessed changes in oxidative status of ram semen supplemented with different antioxidants.

Besides spermatozoa themselves, leukocytes presented in semen are second potential source of ROS, which play an important role during microbial phagocytosis. In comparison to human semen, which commonly contains leukocytes, normal semen of most mammalian species does not contain significant numbers of leukocytes [27]. Therefore, any increased content of leukocytes in ram semen may indicate hidden inflammation in the male reproductive system. Here, we used monoclonal antibodies specific against ram leukocytes (CD18) and monocytes (CD14) [64] to monitor leukocyte content in semen using flow cytometry. This method, which represents a simple and rapid way without preliminary semen purification procedures, was used previously in humans [29] and now, for the first time, also in rams. A significantly increased number of leukocytes, but not monocytes, was detected in Group 2 with poor viability compared to Group 1 (Figure 6). Similarly, increased number of leukocytes, though analyzed by microscopic observation, was negatively correlated with plasma membrane integrity (viability) of ram spermatozoa samples in our previous study [28]. Increased content of ROS as well as leukocytes have been associated with significant damage of sperm DNA [65]. However, we did not observe significant differences between Group 1 and Group 2 in the proportion of sperm with fragmented DNA or in the content of immature spermatozoa using acridine orange (AO) staining procedure (Figure 6). On the contrary, in our previous study, a significant positive correlation was found in ram semen samples between sperm DNA fragmentation and leukocyte content as well as ROS production [28]. Nevertheless, different methods for the evaluation of chromatin status were used in these studies. Furthermore, the AO staining and flow cytometry has been already previously used to assess chromatin integrity in rams or other animal species [66,67].

**Figure A1:**
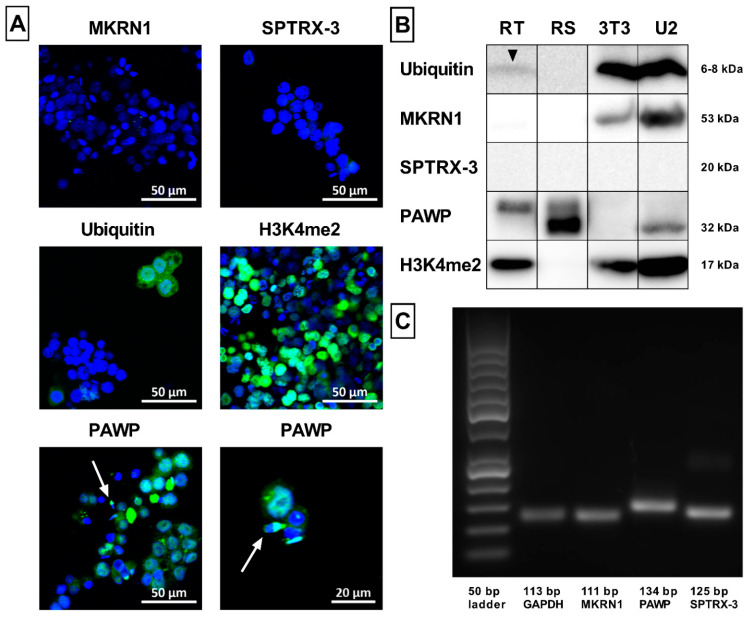
Verification of antibody specificity and expression of selected markers on protein and mRNA level. Illustrative images from confocal microscopy (Zeiss LSM 700; magnification at 200×) showing the staining of ram testis samples by different antibodies (green) and DAPI (blue: nucleus). Antibodies against MKRN1 and SPTRX-3 were found to be nonspecific for ram cells, whereas ubiquitin, PAWP and H3K4me2 antibodies were specific for ram cells. H3K4me2 antibody specifically stain cell nucleus. PAWP antibody detected protein in ram testis cells and spermatozoa (white arrows) (**A**). Comparative Western blot analysis of different cell sample types. Ubiquitin antibody labeled specific protein in ram testis (black arrowhead) as well as PAWP and H3K4me2 antibodies. Only PAWP antibody strongly detected specific protein in randomly selected ram sperm sample. MKRN1 and SPTRX-3 antibodies did not label specific protein neither in ram spermatozoa, nor even in ram testis. RT—ram testis, RS—ram spermatozoa, 3T3—mouse embryonic fibroblasts NIH/3T3, U2—human osteosarcoma cell line U-2 OS (**B**). RT-PCR analysis showing positive expression of selected biomarkers (MKRN1, SPTRX-3 and PAWP) in ram testis sample. GAPDH—housekeeping gene serving as internal control (**C**).

Recent studies reported novel interesting sperm markers not previously studied in ram, which might indicate poor semen quality or even infertility of studied males, such as ubiquitination of the plasma membrane of abnormal spermatozoa in the testis [32] or intracellular stress-induced aggregates of damaged and ubiquitinated proteins, aggresomes (AGG), which are most probably of spermiogenic origin [38]. The ubiquitinated defective spermatozoa can be detected in ejaculated semen using specific antibody against ubiquitin and flow cytometry, as was reported in bull [34,68], stallion [32], pig [69], or using fluorescence microscopy as reported in gaur, buffalo, human or even rhesus monkey [70]. On the other hand, as far as we know, there is no other study focusing on the flow-cytometric assessment of ubiquitinated spermatozoa in ram, probably due to the unavailability of ubiquitin (UBQ) antibodies validated for ram cells or tissue. Here, a clone of UBQ antibody (P4G7-H11) specific, according to producer, for several animal species (ram is not mentioned) was used. We did not observe increased number of ubiquitinated spermatozoa either in Group 1, or Group 2 (Figure 7). However, to fully validate the possible cross-reactivity of the used UBQ antibody, we analyzed the specificity of this antibody for ram by additional confocal microscopy and Western blot analysis of ram testis cells (Appendix A; Figure A1). According to this, specific fluorescent signal and specific protein were detected in ram testis samples using both microscopic and Western blot analyses, respectively, thus confirming the specificity of flow-cytometric analysis. Although no differences in the presence of ubiquitin on the sperm plasma membrane were found between analyzed groups of ram semen with different viability in this study, it may be important to monitor this marker because very recently, it has been reported that high ubiquitin levels in spermatozoa positively correlate with poor freezing ability of stallion semen [71]. On the other hand, significantly higher contents of AGG were observed in Group 2 compared to Group 1 (Figure 7), which clearly correlates with the poor quality of semen samples in Group 2. The differences between UBQ positivity and content of aggresomes in Group 2 might be most likely explained by the different localization of analyzed markers and different staining procedure of ram spermatozoa. In case of UBQ staining, spermatozoa were just fixed without subsequent permeabilization to detect sperm plasma membrane ubiquitination and avoid staining of intrinsic ubiquitinated proteins [70], while for AGG detection, ram spermatozoa were fixed and permeabilized to reach intrinsic protein aggregates. Moreover, our data confirmed that increased AGG content is associated with poor sperm quality, as reported in bull [72] or pig [73].

Besides UBQ and AGG, as potential new ram sperm biomarkers, we studied also MKRN1, SPTRX-3, PAWP and H3K4me2, which have not been reported yet in ram spermatozoa. Expression of MKRN1, a member of spermatogenic genes [74], was found in boar round spermatids [75]. However, an increased accumulation of MKRN1 in defective bovine and human spermatozoa was also observed (Sutovsky; unpublished data), thus making MKRN1 a potential candidate of male infertility. Due to unavailability of ram-specific MKRN1 antibody, a clone of anti-human antibody (OTI2C8) was tested in this study. Because very low positivity and no differences were observed between analyzed semen samples in Group 1 and Group 2 (Figure 8), we concluded that either ram spermatozoa used in the experiments did not express MKRN1, or the used antibody did not cross-react with ram specimen. We, therefore, further explored the specificity of this antibody for ram spermatozoa and testis by confocal microscopy and Western blot analyses (Appendix A; Figure A1) and found this antibody to be nonspecific for ram. On the other hand, we noticed MKRN1 mRNA expression in ram testis (Appendix A; Figure A1) or even in ram spermatozoa previously [76].

The same goes for the expression of SPTRX-3 in ram spermatozoa. The used SPTRX-3 antibody, specific to human, mouse and rat (according to the producer), did not provide relevant fluorescent positivity in ram semen samples of both groups assessed by flow cytometry. Moreover, very low presence of positive spermatozoa was noticed under microscope (Figure 8), which were most probably nonspecifically labeled because SPTRX-3 was found to be located exclusively in round spermatids or superfluous midpiece cytoplasm of defective spermatozoa [38]. Further analyses did not reveal specific staining of ram testis cells or detection of specific SPTRX-3 protein by Western blot. On the contrary, mRNA expression of SPTRX-3 in ram testis was again confirmed by PCR analysis (Appendix A; Figure A1), thus indicating that SPTRX-3, as well as MKRN1 expression, should be assessed also in ram testis and defective spermatozoa using species-specific or cross-reactive antibodies. Moreover, it was demonstrated that higher levels of SPTRX-3 in semen correlate with male infertility [37,38]; therefore, SPTRX-3 might be an important quality indicator of individual ram semen intended for the preservation of genetic resources.

Contrary to MKRN1 and SPTRX-3 antibodies, antibodies against PAWP and H3K4me2 seem to specifically cross react with ram spermatozoa. Three ram sperm populations can be clearly distinguished, according to PAWP positivity, as the spermatozoa with low, moderate and high PAWP level (Figure 8), similar to what was described previously on bovine spermatozoa [72]. It was observed that high or even low level of PAWP is associated with poor sperm quality and fertility in bull and human [72,77]. Similarly, we observed increased number of ram spermatozoa with high PAWP content in Group 2 compared to Group 1 (Figure 8). On the other hand, moderate PAWP level negatively correlated with aggresome content in bull spermatozoa [72]. This also agree with our findings because low AGG positivity was observed in Group 1, where the majority of ram spermatozoa exhibited moderate expression of PAWP. The specific staining of ram spermatozoa with the used PAWP antibody was confirmed also by confocal microscopy (Figure 8). The same staining pattern and PAWP localization was observed in the post-acrosomal sperm sheath of bull, pig, rabbit, rhesus monkey [78] and human [77]. Furthermore, confocal microscopy revealed positive staining of ram testis cells, and specific PAWP proteins were detected in ram sperm and testis samples using this antibody in Western blot analysis. The PAWP expression on mRNA level was also demonstrated in ram testis (Appendix A; Figure A1) as well as in ram spermatozoa in our previous study [76].

The last potential biomarker of ram sperm quality tested in this study was H3K4me2. This epigenetic marker is well studied in spermatozoa as candidate of male infertility [39]. In general, remodeling of chromatin during spermatogenesis is quite susceptible to its environment, and increased oxidative stress is the main reason for compromised DNA integrity [79,80,81]. For this reason, finding of an epigenetic marker associated with ram semen quality is of a great interest. It was observed that increased levels of H3K4me2 in human semen samples with poor quality were significantly related to sperm chromatin immaturity (% HDS) and negatively correlated with sperm motility, concentration and activity of mitochondria [39]. Here, we did not observe differences in H3K4me2 level between analyzed groups of ram spermatozoa with different viability and quality (Figure 8). However, we also found no differences in chromatin integrity of ram spermatozoa belonging to Group 1 and Group 2 (Figure 6). Thus, significant chromatin aberrations were probably lacking in the analyzed ram semen samples. Nevertheless, the specificity of used H3K4me2 antibody was again confirmed both by confocal microscopy of ram spermatozoa (Figure 8) and testis or by Western blot analysis (Appendix A; Figure A1).

## 4. Materials and Methods

### 4.1. Animals and Semen Collection

In this study, sexually mature (2.5–5 years old) and clinically healthy rams of the Native Wallachian (*n* = 4) and Improved Wallachian (*n* = 2) sheep breeds were used. Rams were kept under external conditions in individual stalls at a breeding facility (NPPC, RIAP Nitra, Lužianky, Slovak Republic) and fed with hay bales and oats; water and mineral salts were supplied ad libitum. Semen samples (*n* = 58) were collected twice a week by electro-ejaculation and immediately transported to the laboratory throughout the whole study (September–November), as described previously [82]. The study was conducted during the breeding season because it was reported that seasonality can influence ram reproductive organs [83] and reproductive performance in general.

### 4.2. Computer-Assisted Semen Analysis (CASA)

Sperm motility and concentration were assessed using CASA system with Sperm Vision™ software (MiniTube, Tiefenbach, Germany) as described previously [82]. Briefly, each fresh semen sample was analyzed for average concentration (10^9^ spermatozoa/mL, percentage of totally motile spermatozoa (motility > 5 μm/s) and percentage of progressively motile spermatozoa (motility > 20 μm/s). The concentration of sperm samples was not standardized before its measurement, in order to assess sperm concentration under physiological (native) conditions immediately after collection in fresh (neat) semen. At first, fresh ram semen was diluted by saline (0.9% NaCl; Braun, Melsungen, Germany) at the ratio (1:40). If necessary, higher, or lower dilution rate was used to obtain optimal sperm concentration for CASA measurement. Afterwards, 10 μL of prediluted semen sample was transferred to Makler counting chamber (Sefi Medical Instruments, Haifa, Israel) and analyzed with SpermVision^TM^ software under AxioScope A1 light microscope (Carl Zeiss Slovakia, Bratislava, Slovakia). Sperm motility and concentration were automatically analyzed in seven microscopic view fields at 60 frames per second within less than one minute. The CASA system automatically controls the sperm concentration in all measured fields. If the concentration for an analysis is not statistically valid, the system will alert the technician, and analyses must be repeated. Moreover, the fully experienced technician usually repeats the analyses by himself, if the measurement seems doubtful.

### 4.3. Experimental Design and Flow-Cytometric Analyses

Sperm samples (aliquots from each ram semen) were diluted to the concentration of 1 × 10^6^ spermatozoa in a phosphate buffered saline (PBS, Ca- and Mg- free; Biosera, Nuaille, France) or other specific buffer (e.g., Annexin V Binding Buffer) and incubated with selected chemicals, which specifically identify common physiological sperm characteristics as viability and apoptosis, acrosomal status, capacitation, mitochondrial activity, generation of reactive oxygen species (ROS) and chromatin status. Moreover, in this study, we compared several probes for the detection of apoptotic-like changes, integrity of acrosome, sperm capacitation, activity of mitochondria and ROS generation in order to choose more suitable markers for ram sperm quality analysis. In addition, the increased occurrence of leukocytes in the semen samples and expression of novel fertility-related biomarkers, such as ubiquitination and formation of aggresomes, overexpression of MKRN1, SPTRX-3 and PAWP proteins or histone modification (H3K4me2), were also analyzed by flow cytometry. Samples were analyzed immediately after staining and/or washing procedure using FACSCalibur flow cytometer (BD Biosciences, San Jose, CA, USA) equipped with a 488 nm argon ion laser and red-diode (635 nm) laser. Fluorescent signals were acquired by Cell Quest Pro ™ software (BD Biosciences, San Jose, CA, USA) in green FL1 channel using 530/30 nm band pass filter, orange FL2 channel using 585/42 nm band pass filter, red FL3 channel using 670 nm long pass filter and/or far-red FL4 channel using 661/16 nm band pass filter. Calibration of the instrument was performed periodically using standard calibration beads (BD CaliBRITE™; BD Biosciences, San Jose, CA, USA). At least 10,000 events (spermatozoa) were acquired for each sample using log scale and low flow rate (about 600–1000 events/s) unless otherwise stated. Unstained samples or samples stained with secondary antibodies were used as control samples in order to gate the positive cells according to the increased fluorescent intensity. Obtained flow-cytometric data were evaluated using FlowJo™ v10.8.1 Software (BD Biosciences, San Jose, CA, USA).

The motility of spermatozoa is one of the basic parameters defining the overall quality of analyzed semen samples. However, our preliminary experiments showed that the motility of ram spermatozoa do not always correlate with their viability. On the other hand, a good sperm viability is prerequisite for higher cryosurvival rates of frozen-thawed spermatozoa. Therefore, in this study, we divided all analyzed semen samples into two groups according to their viability: more than 60% of viable spermatozoa (Group 1) and less than 60% of viable spermatozoa (Group 2). The assessed parameters of sperm quality by CASA and flow cytometry were then compared between these two groups.

#### 4.3.1. Viability and Apoptosis

The viability of spermatozoa was assessed using SYBR-14 [7], a membrane-permeant nucleic acid green fluorescent dye (LIVE/DEAD^®^ Sperm Viability Kit; Thermo Fisher Scientific, Waltham, MA, USA) and DRAQ7, a far-red fluorescent nucleic acid dye (BioStatus Limited, Shepshed, UK), which stains nuclei of dead or membrane-compromised cells. Briefly, 1 × 10^6^ spermatozoa were incubated with 2.5 µL of SYBR-14 (at final concentration of 100 nM) for 10 min in the dark at 37 °C. The washing step was omitted, and samples were immediately co-stained with ready-to-use DRAQ7 dye (at final concentration of 3 µM) for 10 min in the dark at room temperature (RT). Afterwards, samples without further washing were analyzed by flow cytometer. The proportion (%) of spermatozoa positive for SYBR-14 but negative for DRAQ7 was considered as proportion of live (SYBR-14^+^/DRAQ7^−^), while SYBR-14^+^/DRAQ7^+^ and SYBR-14^−^/DRAQ7^+^ spermatozoa were considered as dead spermatozoa (moribund and necrotic spermatozoa, respectively; Figure 1A).

To observe the apoptotic-like changes in ram spermatozoa, four green fluorescent probes were used, each with specific binding pattern. Annexin V (AnV) was used to detect changes in the plasma membrane (phosphatidylserine translocation) of ram spermatozoa [10]. Briefly, 1 × 10^6^ spermatozoa were diluted in 98 µL of 1× Annexin V Binding Buffer and incubated with 2 µL of Annexin V-FITC (both are components of Annexin V Apoptosis Detection Kit; Canvax, Cordoba, Spain) for 15 min in the dark at RT. Samples were washed in 1× Annexin V Binding Buffer and centrifuged at 600× *g* and 20 °C for 5 min. Subsequently, spermatozoa were resuspended in 200 µL of 1× Annexin V Binding Buffer and stained with ready-to-use DRAQ7 dye, as stated above. Unwashed samples were subjected to the flow-cytometric analysis, in which the proportion (%) of spermatozoa positive for Annexin V (AnV^+^/DRAQ7^−^ and AnV^+^/DRAQ7^+^) was considered as a proportion of apoptotic-like spermatozoa (Figure 1A).

YO-PRO-1 nuclear green dye (Thermo Fisher Scientific, Waltham, MA, USA) was used to detect apoptotic-like changes in ram spermatozoa. Semen samples (1 × 10^6^ spermatozoa) were diluted in 500 µL of PBS and incubated with 0.5 µL of YO-PRO-1 (at final concentration of 100 nM) [84] for 15 min in the dark at RT. Samples were washed in PBS by centrifugation (600× *g*, 20 °C, 5 min), stained with ready-to-use DRAQ7 dye, as stated above, and analyzed by flow cytometer. The proportion (%) of spermatozoa positive for YO-PRO-1 (YO-PRO-1^+^/DRAQ7^−^ and YO-PRO-1^+^/DRAQ7^+^) was considered as proportion of apoptotic-like spermatozoa (Figure 1A).

To detect activity of caspases, two probes were used in the experiments. The first one, green FLICA reagent (Vybrant™ FAM Poly Caspases Assay Kit; Thermo Fisher Scientific, Waltham, MA, USA), detects active poly caspases [12]. Briefly, 1 × 10^6^ spermatozoa were diluted in 300 µL of PBS and incubated with 5 µL of 5X FLICA working solution for 30 min in the dark at 37 °C. The working solution was prepared by diluting 150X FLICA stock solution in PBS at the ratio of 1:30. After incubation, samples were washed twice (600× *g*, 20 °C, 5 min), stained with ready-to-use DRAQ7 dye, as stated above and analyzed by flow cytometer. The proportion (%) of spermatozoa positive for FLICA (FLICA^+^/DRAQ7^−^ and FLICA^+^/DRAQ7^+^) was considered as proportion of apoptotic-like spermatozoa (Figure 1A).

The second caspase detecting reagent, Caspase 3/7 (CellEvent™ Caspase-3/7 Green Flow Cytometry Assay Kit; Thermo Fisher Scientific, Waltham, MA, USA), specifically recognizes active caspase-3 and caspase-7 proteins [85]. Semen samples (1 × 10^6^ spermatozoa) were diluted in 500 µL of PBS and incubated with 0.5 µL of Caspase 3/7 for 30 min in the dark at 37 °C. The washing step was omitted, samples were immediately co-stained with ready-to-use DRAQ7 dye, as stated above, and analyzed by flow cytometer. The proportion (%) of spermatozoa positive for Caspase 3/7 (Caspase 3/7^+^/DRAQ7^−^ and Caspase 3/7^+^/DRAQ7^+^) was considered as a proportion of apoptotic-like spermatozoa (Figure 1A).

#### 4.3.2. Acrosomal Status

The integrity of acrosome was inspected using two different fluorescent probes: PNA (peanut agglutinin) and LCA (*Lens culinaris* agglutinin), and a specific antibody against GAPDHS (sperm-specific glyceraldehyde phosphate dehydrogenase), which is an intra-acrosomal protein. One µL of PNA working solution (Alexa Fluor 488 conjugate; Thermo Fisher Scientific, Waltham, MA, USA) was incubated with 1 × 10^6^ spermatozoa diluted in 200 µL of PBS for 15 min in the dark at RT. PNA working solution (at concentration of 0.5 mg/mL) [86] was prepared by dissolving of the protein (1 mg/mL) in 2 mL of deionized water. After incubation, samples were washed (600× *g*, 20 °C, 5 min), stained with ready-to-use DRAQ7 dye, as stated above, and analyzed by flow cytometer. The proportion (%) of spermatozoa positive for PNA (PNA^+^/DRAQ7^−^ and PNA^+^/DRAQ7^+^) was considered as proportion of acrosome-damaged spermatozoa (Figure 3A).

Ten µL of GAPDHS antibody [15] conjugated with FITC (clone Hs-8; EXBIO Praha, Vestec, Czech Republic) were incubated with 1 × 10^6^ spermatozoa diluted in 50 µL of PBS for 15 min in the dark at RT according to the producer’s manual. After incubation, samples without further washing were adjusted to the final volume of 200 µL with PBS, stained with ready-to-use DRAQ7 dye, as stated above, and analyzed by flow cytometer. The proportion (%) of spermatozoa positive for GAPDHS (GAPDHS^+^/DRAQ7^−^ and GAPDHS^+^/DRAQ7^+^) was considered as a proportion of acrosome-damaged spermatozoa (Figure 3A).

One µL of LCA lectin conjugated with red rhodamine (at final concentration of 10 µg/mL; Vector Laboratories, Burlingame, CA, USA) in combination with 0.5 µL SYTOX^®^ Green dead cell stain (at final concentration of 30 nM; Thermo Fisher Scientific, Waltham, MA, USA) were incubated with 1 × 10^6^ spermatozoa diluted in 500 µL of PBS for 15 min in the dark at RT. After incubation, samples were washed (600× *g*, 20 °C, 5 min) and analyzed by flow cytometer. The proportion (%) of spermatozoa positive for LCA (LCA^+^/SYTOX Green^−^ and LCA^+^/SYTOX Green^+^) was considered as a proportion of acrosome-damaged spermatozoa (Figure 3A).

#### 4.3.3. Sperm Capacitation Status

Capacitation of ram spermatozoa was evaluated using two different fluorescent probes: red dye merocyanine 540 (M540; Thermo Fisher Scientific, Waltham, MA, USA), which detects alterations in the lipid distribution within the sperm plasma membrane, and FLUO-4 AM, specific Ca^2+^ green fluorescent probe (FLUO-4; Thermo Fisher Scientific, Waltham, MA, USA). M540 dye (at final concentration of 2.7 µM) [18] in combination with SYTOX^®^ Green dead cell stain (30 nM), as stated above, were incubated with 1 × 10^6^ spermatozoa diluted in 500 µL of PBS for 15 min in the dark at RT. After incubation, samples were washed (600× *g*, 20 °C, 5 min) and analyzed by flow cytometer. The proportion (%) of spermatozoa positive for M540 (M540^+^/SYTOX Green^−^ and M540^+^/SYTOX Green^+^) was considered as proportion of capacitated spermatozoa (Figure 3D).

FLUO-4 dye [20] (at final concentration of 100 nM) was incubated with 1 × 10^6^ spermatozoa diluted in 500 µL of PBS for 20 min in the dark at 37 °C. Afterwards, samples were washed (600× *g*, 20 °C, 5 min), stained with ready-to-use DRAQ7 dye, as stated above, and analyzed by flow cytometer. The proportion (%) of spermatozoa positive for FLUO-4 (FLUO-4^+^/DRAQ7^−^ and FLUO-4^+^/DRAQ7^+^) was considered as a proportion of capacitated spermatozoa (Figure 3D).

#### 4.3.4. Mitochondrial Activity

The activity of mitochondria was assessed through the mitochondrial membrane potential (MMP) using three different fluorescent probes: MitoTracker^®^ Green FM (MT Green; Thermo Fisher Scientific, Waltham, MA, USA), Rhodamine 123 (Rh123; Merck, Darmstadt, Germany) and eBioscience™ JC-1 Mitochondrial Membrane Potential Dye (JC-1; Thermo Fisher Scientific, Waltham, MA, USA) [22]. Briefly, 1 × 10^6^ spermatozoa diluted in 500 µL of PBS were incubated with MT Green dye (at final concentration of 300 nM) in the dark at 37 °C for 10 min. After incubation, samples were washed (600× *g*, 20 °C, 5 min), stained with ready-to-use DRAQ7 dye, as stated above, and analyzed by flow cytometer. The proportion (%) of spermatozoa positive for MT Green (MT Green^+^/DRAQ7^−^) was considered as proportion of spermatozoa with high MMP (Figure 4A).

Rh123 green dye (at final concentration of 10 ng/mL) was added to 1 × 10^6^ spermatozoa diluted in 500 µL of PBS and incubated for 10 min in the dark at 37 °C. Afterwards, samples were washed (600× *g*, 20 °C, 5 min), stained with ready-to-use DRAQ7 dye, as stated above, and analyzed by flow cytometer. The proportion (%) of spermatozoa positive for Rh123 (Rh123^+^/DRAQ7^−^) was considered as a proportion of spermatozoa with high MMP (Figure 4A).

JC-1 dye (at final concentration of 50 ng/mL) was added to 1 × 10^6^ spermatozoa diluted in 500 µL of PBS and incubated for 10 min in the dark at 37 °C. Afterwards, samples were washed twice in PBS (600× *g*, 20 °C, 5 min) and analyzed by flow cytometer. The proportion (%) of spermatozoa positive for JC-1 orange aggregates was considered as proportion of spermatozoa with high MMP (Figure 4A).

#### 4.3.5. Generation of Reactive Oxygen Species (ROS)

To measure the production of ROS in ram semen samples, four different fluorescent probes were used: (1) chloromethyl derivative of H_2_DCFDA, a green dye nonspecifically indicating presence of intracellular ROS (CM-H_2_DCFDA; Thermo Fisher Scientific, Waltham, MA, USA); (2) dihydroethidium (hydroethidine), a red superoxide indicator (DHE; Thermo Fisher Scientific, Waltham, MA, USA); (3) MitoSOX™ Red mitochondrial superoxide indicator (MitoSOX; Thermo Fisher Scientific, Waltham, MA, USA), and (4) BODIPY™ 581/591 C11, a green lipid peroxidation sensor (BODIPY; Thermo Fisher Scientific, Waltham, MA, USA).

Briefly, 1 × 10^6^ spermatozoa diluted in 500 µL of PBS were incubated with CM-H_2_DCFDA probe [23] (at final concentration of 500 nM) for 30 min in the dark at 37 °C. After incubation, samples were washed (600× *g*, 20 °C, 5 min), stained with ready-to-use DRAQ7 dye, as stated above, and analyzed by flow cytometer. The proportion (%) of spermatozoa positive for CM-H_2_DCFDA (CM-H_2_DCFDA^+^/DRAQ7^−^ and CM-H_2_DCFDA^+^/DRAQ7^+^) was considered as a proportion of ROS-positive spermatozoa (Figure 5A).

DHE dye (at final concentration of 2 µM) in combination with SYTOX^®^ Green dead cell stain (30 nM) [25], as stated above, were added to 1 × 10^6^ spermatozoa diluted in 500 µL of PBS and incubated for 10 min in the dark at 37 °C. After incubation, samples were washed (600× *g*, 20 °C, 5 min) and analyzed by flow cytometer. The proportion (%) of spermatozoa positive for DHE (DHE^+^/SYTOX Green^−^ and DHE^+^/SYTOX Green^+^) was considered as a proportion of ROS-positive spermatozoa (Figure 5A).

MitoSOX probe (at final concentration of 500 nM) in combination with SYTOX^®^ Green dead cell stain (30 nM) [25], as stated above, were added to 1 × 10^6^ spermatozoa diluted in 1000 µL of PBS with calcium and magnesium (PBS Ca^2+^; Biosera, Nuaille, France) and incubated for 15 min in the dark at 37 °C. After incubation, samples were washed twice in PBS Ca^2+^ (600× *g*, 20 °C, 5 min) and analyzed by flow cytometer. The proportion (%) of spermatozoa positive for MitoSOX (MitoSOX^+^/SYTOX Green^−^ and MitoSOX^+^/SYTOX Green^+^) was considered as proportion of ROS-positive spermatozoa (Figure 5A).

BODIPY probe [26] (at final concentration of 200 nM) was added to 1 × 10^6^ spermatozoa diluted in 500 µL of PBS and incubated for 30 min in the dark at 37 °C. Afterwards, samples were washed (600× *g*, 20 °C, 5 min), stained with ready-to-use DRAQ7 dye, as stated above, and analyzed by flow cytometer. The proportion (%) of spermatozoa positive for BODIPY (BODIPY^+^/DRAQ7^−^ and BODIPY^+^/DRAQ7^+^) was considered as a proportion of ROS-positive spermatozoa (Figure 5A).

#### 4.3.6. Chromatin Status

To detect damaged sperm chromatin, an acridine orange dye (AO, 10 mg/mL in water; Thermo Fisher Scientific, Waltham, MA, USA) was used. AO dye changes from green (dsDNA) to red fluorescent color (ssDNA) according to the degree of DNA denaturation [30]. Briefly, 2 × 10^6^ spermatozoa, diluted in 200 µL of PBS, were incubated with 400 µL of ice-cold acid-detergent solution (pH 1.2; 0.08 N HCl, 0.15M NaCl, 0.1% Triton-X 100) for 30 s in order to denature sperm DNA. Afterwards, samples were stained immediately with 1.2 mL of ice-cold staining solution (pH 6.0; 0.1 M citric acid, 0.2 M Na_2_PO_4_, 1 mM EDTA, 0.15 M NaCl) containing AO (at final concentration of 6 µg/mL) for 3 min on ice in the dark. Stained samples were then immediately analyzed, without washing, using flow cytometer in a linear scale of green FL1 channel and red FL3 channel at the flow rate of 300 cells/s. Each sample was measured twice. The tube containing detergent and staining solution, at the same ratio as in the experimental sample, was used to equilibrate the flow cytometer at least 5 min before acquiring the experimental sample. The proportion (%) of spermatozoa bearing loose chromatin (shift to red fluorescence) was considered as a proportion of spermatozoa with DNA fragmentation (% SDF), and immature spermatozoa (high green fluorescence) were classified as spermatozoa with high DNA stainability (% HDS; Figure 6A).

#### 4.3.7. Occurrence of Leukocytes

Leukocytes presented in the ram semen samples were evaluated using monoclonal antibodies specific against CD18 (all leukocytes) and CD14 (monocytes/macrophages) membrane markers [64]. Briefly, 1 × 10^6^ spermatozoa diluted in 50 µL of PBS were co-stained with 0.5 µL of purified antibodies CD18 (clone BAQ30A, IgG1) and CD14 (clone CAM66A, IgM; both from WSU, Pullman, WA, USA) for 15 min on ice in the dark. After washing in PBS (600× *g*, 20 °C, 5 min), samples were incubated with 1 µL of rat anti-mouse IgG1-FITC (clone M1-14D12) and rat anti-mouse IgM-PE secondary antibodies (clone II/41; both from Thermo Fisher Scientific, Waltham, MA, USA) for 15 min on ice in the dark. Afterwards, samples were washed, stained with ready-to-use DRAQ7 dye, as stated above, in order to exclude the dead cells from the analysis and analyzed by flow cytometer. The proportion (%) of cells positive for CD18 (CD18^+^/CD14^−^ and CD18^+^/CD14^+^) was classified as leukocytes; double positive cells (CD18^+^/CD14^+^) were classified as monocytes/macrophages (Figure 6C).

#### 4.3.8. Ubiquitination and Formation of Aggresomes

To detect defective ubiquitinated spermatozoa and aggregates of ubiquitinated proteins (aggresomes) in ram semen samples, purified mouse anti-ubiquitin monoclonal antibody (UBQ; clone P4G7-H11, IgG1) and PROTEOSTAT^®^ Aggresome detection kit (both from Enzo Life Sciences, Farmingdale, NY, USA) were used [72]. Briefly, 2 × 10^6^ spermatozoa were fixed in IC Fixation Buffer (Thermo Fisher Scientific, Waltham, MA, USA) for 20 min at RT. Afterwards, samples were washed in PBS (1000× *g*, 4 °C, 5 min) and blocked with heat-inactivated sheep serum prepared in our laboratory from ovine peripheral blood. After blocking, samples were diluted in 50 µL of washing and staining buffer (WSB; PBS containing 0.5% BSA and 0.05% sodium azide (NaN3)) and incubated with 5 µL of UBQ antibody in the dark at 4 °C overnight. Then, samples were washed in WSB (1000× *g*, 4 °C, 5 min) and incubated with 0.5 µL of goat anti-mouse IgG-FITC polyclonal secondary antibody (STAR117F; Bio-Rad, Hercules, CA, USA) for 15 min on ice in the dark. After washing in WSB (1000× *g*, 4 °C, 5 min), samples were analyzed by flow cytometer. The proportion (%) of spermatozoa positive for ubiquitin was classified as defective spermatozoa (Figure 7A).

PROTEOSTAT^®^ Aggresome detection kit was applied to ram semen samples according to the producer’s manual with some modifications. Briefly, 2 × 10^6^ spermatozoa were fixed in IC Fixation Buffer, as stated above, and washed in PBS (1000× *g*, 4 °C, 5 min). Samples were then incubated with permeabilizing solution (PBS containing 0.1% Triton X-100) for 30 min on ice. Afterwards, samples were washed twice in PBS (1000× *g*, 4 °C, 5 min) and incubated in 500 µL of staining solution containing PBS and freshly diluted (100,000-fold) PROTEOSTAT^®^ Aggresome detection kit for 30 min in the dark at RT. At last, samples without washing were analyzed by flow cytometer in red FL3 channel. The proportion (%) of spermatozoa positive for aggresomes was classified as defective spermatozoa (Figure 7A).

#### 4.3.9. Intracellular Fertility Biomarkers

To assess possible expression of novel fertility related biomarkers, ram spermatozoa were stained with purified mouse monoclonal antibody against makorin ring finger protein-1 (MKRN1, clone OTI2C8, IgG2b; Thermo Fisher Scientific, Waltham, MA, USA) and rabbit polyclonal (IgG) antibodies against spermatid-specific thioredoxin-3 (SPTRX-3, known also as thioredoxin domain-containing protein 8 (TXNDC8); EMZ003; Kerafast, Boston, MA, USA) and against post-acrosomal WW-domain binding protein (PAWP, known also as WW domain binding protein 2 N-Terminal Like (WBP2NL); 22587-1-AP; Proteintech Group, Rosemont, IL, USA), and rabbit monoclonal antibody against histone H3 dimethylated on lysine K4 (H3K4me2, clone Y47, IgG; Abcam, Cambridge, UK).

Briefly, 2 × 10^6^ spermatozoa were fixed and permeabilized as stated before. Samples were diluted in 50 µL of WSB and incubated separately either with 2 µL of MKRN1 antibody, 1 µL of SPTRX-3 antibody, 1 µL of PAWP antibody or 1 µL of H3K4me2 antibody in the dark at 4 °C overnight. Then, samples were washed in WSB (1000× *g*, 4 °C, 5 min.) and incubated with goat polyclonal secondary antibodies: 0.5 µL of anti-mouse IgG-FITC (STAR117F; Bio-Rad, Hercules, CA, USA) in case of MKRN1 and 0.5 µL of anti-rabbit IgG-FITC (405002; Bio-Rad, Hercules, CA, USA) in case of SPTRX-3, PAWP and H3K4me2 for 15 min on ice in the dark. After washing in WSB (1000× *g*, 4 °C, 5 min), samples were analyzed by flow cytometer. The proportion (%) of spermatozoa positive for MKRN1 and SPTRX-3 was classified as defective spermatozoa (Figure 8A). Using PAWP antibody, spermatozoa with low, moderate and high PAWP content can be distinguished, while spermatozoa with high PAWP content were classified as defective spermatozoa (Figure 8A) [72]. Similarly, sperm samples with higher mean fluorescence intensity (MFI) values of H3K4me2 were classified as defective spermatozoa (Figure 8C,D). The final value for MFI of H3K4me2 was obtained after subtracting the MFI of control sample stained only with secondary antibody from the signal (MFI) of the experimental sample. Moreover, we analyzed the specificity of used antibodies (UBQ, MKRN1, SPTRX-3, PAWP and H3K4me2) for ram spermatozoa and testes by additional confocal microscopy and Western blot analysis (Appendix A; Figure A1).

### 4.4. Confocal Microscopy

To check the specificity of flow-cytometric staining, a microscopic assessment of selected sperm samples was performed. Briefly, selected sperm samples were labeled using all above-mentioned fluorescent probes, reagents and antibodies. An aliquot of stained sample (2 µL) was mixed with 2 µL of the VECTASHIELD anti-fade mounting medium with DAPI (Vector Laboratories, Burlingame, CA, USA), dropped onto the microscope slide and mounted with a coverslip. Prepared samples were analyzed using Zeiss LSM 700 laser scanning confocal microscope (Carl Zeiss Slovakia, Bratislava, Slovakia) equipped with a blue 405 nm, green 488 nm and red 555 nm laser, and T-PMT (photomultiplier for transmitted light) for acquiring sample images in bright-field or differential interference contrast (DIC).

### 4.5. Statistical Analysis

Data obtained from flow-cytometric analyses (27 samples in Group 1 and 31 samples in Group 2) were evaluated using GraphPad Prism version 9.3.1 for Windows (GraphPad Software, San Diego, CA, USA) with two-way ANOVA followed by Sidak test for multiple comparisons. Results are expressed as the mean ± SD. *p*-values at *p* < 0.05 were considered as statistically significant.

## 5. Conclusions

The proposed multiparametric analysis of ram spermatozoa provides quite rapid and complex screening of semen quality using flow cytometry in comparison to time-consuming microscopic assessment. Several important sperm attributes, such as membrane integrity, apoptosis, mitochondrial activity, oxidative stress, aggresome formation, etc., might be evaluated using the above-mentioned specific fluorescent probes in ram semen samples. Moreover, several antibodies for the detection of new biomarkers (ubiquitin, PAWP and H3K4me2) associated with sperm quality were successfully validated for ram spermatozoa. However, further study is required in order to find antibodies specific to other possible ram sperm biomarkers, such as MKRN1 and SPTRX-3. At last, using such analysis, an important quality control of semen samples obtained from valuable breeding males can be done prior its further processing such as cryopreservation, which can be crucial for successful cryosurvival rates. Moreover, several parameters from this analysis (e.g., apoptotic-like markers, ROS, chromatin integrity, etc.) may be involved as criteria for quality control applied to sperm cryopreservation protocols.

## Data Availability

The data presented in this study are available in article.

## Appendix A

### Appendix A.1. Confocal Microscopy

To check the specificity of antibodies (UBQ, MKRN1, SPTRX-3, PAWP and H3K4me2) used for flow-cytometric analyses of ram spermatozoa, a microscopic assessment of ram testis samples was performed. Ram testes were obtained from the local slaughterhouse and transported to our laboratory for further processing. Briefly, after removal of *tunica albuginea* and connective tissue, testes were minced into small fragments and washed with PBS (Ca- and Mg- free; Biosera, Nuaille, France) containing 5% penicillin/streptomycin antibiotics (Thermo Fisher Scientific, Waltham, MA, USA). Testis tissue fragments were incubated at 37 °C for 30 min with collagenase type I (Merck, Darmstadt, Germany). The enzymatic solution was neutralized with αMEM culture medium containing 10% FBS (fetal bovine serum; Merck, Darmstadt, Germany) and centrifuged at 300× *g* for 5 min. Subsequently, testis fragments were incubated at 37 °C for 30 min with 0.25% Trypsin-EDTA solution (Thermo Fisher Scientific, Waltham, MA, USA), washed in αMEM medium, as previously mentioned (300× *g*, 5 min) and filtered through a 100 µm filter to obtain single cell suspension. This heterogeneous mixture of ram testis cells was counted and appropriate number of cells were fixed, permeabilized and stained using primary and secondary antibodies, as mentioned before for ram spermatozoa in Materials and Methods. Labeled samples were mounted on microscopic slides and analyzed using a Zeiss LSM 700 confocal microscope. A positive fluorescent signal was found for UBQ, PAWP and H3K4me2, while no positivity was detected for MKRN1 and SPTRX-3 (Figure A1A).

### Appendix A.2. Western Blot Analysis

To further confirm the specificity of above-mentioned antibodies for ram samples, we performed Western blot analysis of ram testis cells, randomly selected ram sperm samples and two commercial cell lines, U-2 OS (human osteosarcoma cells; ECACC 92022711) and NIH/3T3 (mouse embryonic fibroblasts; ATCC^®^ CRL1658™), which served as internal positive controls for selected antibodies. Briefly, cell samples were placed on ice for 10 min and then washed twice in PBS (Ca- and Mg- free; Biosera, Nuaille, France) at 139× *g* for 8 min. Subsequently, samples were lysed in 200 μL of 1× SDS sample buffer (pH 6.8; 50 mM Tris-HCl, 2% SDS, 200 mM 2-mercaptoethanol, 0.03% bromophenol blue, 10% glycerol), boiled for 5 min and subjected to SDS-PAGE on 4–15% Mini protean TGX gels (Bio-Rad, Hercules, CA, USA) at constant current (30 mA). Following electrophoresis, proteins were transferred either to an 0.2 μm PVDF membrane (Bio-Rad, Hercules, CA, USA) for ubiquitin and SPTRX-3 proteins, or to 0.45 μm nitrocellulose membrane (for PAWP, H3K4me2 and MKRN1 proteins) using tank transfer for 30 min at constant current (150 mA). After protein transfer, membranes were blocked in 5% nonfat dry milk in Tris-buffered saline-tween buffer (TBS-T; pH 7.5) for 60 min at room temperature and then incubated with the primary antibody in 3% non-fat dry milk in TBS-T at 4 °C overnight.

The dilutions of primary antibodies were as follows: Ubiquitin (1:1000), MKRN1 (1:1000), SPTRX-3 (1:2000), PAWP (1:1000) and H3K4me2 (1:2000). Primary antibody binding sites were labeled by an incubation with a secondary horseradish peroxidase (HRP)-conjugated antibody (sheep anti-mouse or donkey anti-rabbit IgG; 1:5000; GE Healthcare Life Sciences, Amersham, UK) in 3% nonfat dry milk in TBS-T for 60 min at room temperature. Target proteins were revealed using SuperSignal^TM^ West Pico PLUS Chemiluminescent Substrate (Thermo Fisher Scientific, Waltham, MA, USA). The luminescent signals were visualized using a MF-ChemiBIS 3.2. device (DNR Bio Imaging Systems, Neve Jamin, Israel). A specific ubiquitin protein (6–8 kDa) was observed in ram testis, both cell lines, but not in randomly selected ram sperm sample. MKRN1 antibody labeled specific protein (53 kDa) in both cell lines, but not in ram sperm or testis sample. On the other hand, SPTRX-3 antibody did not detect a specific protein in any of the analyzed samples. PAWP antibody labeled specific protein (32 kDa) in ram spermatozoa, thus confirming the strong expression detected using flow cytometry. PAWP protein was detected also in U-2 OS, but not in NIH/3T3 cell line. Interestingly, a specific protein of slightly higher molecular mass was observed in ram testis. The difference in molecular mass of PAWP protein was reported previously between mouse sperm and testis sample as well as among mouse, boar, bovine and human spermatozoa [78]. Thus, the molecular mass of PAWP protein seems to depend on sperm developmental stage as well as on analyzed species. At last, H3K4me2 antibody labeled specific protein (17 kDa) in both cell lines and ram testes, although this protein was not detected in randomly selected ram sperm sample (Figure A1B).

### Appendix A.3. RT-PCR Analysis

Expression of selected biomarkers (MKRN1, SPTRX-3 and PAWP) in ram testis on mRNA level was explored using RT-PCR analysis and specific primers. Briefly, total mRNA was extracted from ram testis cells using TRI Reagent RT (MRC, Cincinnati, OH, USA) according to manufacturer’s instructions. Purity of extracted RNA was determined by 260:280 nm ratio and integrity checked by electrophoresis in 1% agarose gel. RNA samples were treated with the dsDNase (Thermo Fisher Scientific, Waltham, MA, USA) before reverse transcription to destroy contaminating DNA. The first strand cDNA was synthesized using Maxima H Minus First Strand cDNA Synthesis Kit (Thermo Fisher Scientific, Waltham, MA, USA) with 1µg of total RNA, oligo (dT)18 and random hexamer primers in total volume of 20 µL. The reaction was performed at 55 °C for 30 min and terminated at 85 °C for 5 min. PCR was performed in 20 μL reactions consisting of 10 μL PCR Master Mix 2X (Promega, Madison, WI, USA), 1 μL cDNA, 3 mM MgCl_2_, 0.25 μM forward primer and 0.25 μM reverse primer for MKRN1 (5′–3′, AATGCCATCGAGTTTGTTCC; TTGCTCCTTCTCCGTGTCTT; 111 bp), SPTRX-3 (5′–3′, GTTGGCCCAAACTTACCAGA; CGCTCAGGGATCCACTTCTA; 125 bp), PAWP (5′–3′, ATGGCACAAAGAAAGGAACG; TGGTTGTTCAATGGTGCAGT; 134 bp) and GAPDH (5′–3′, TAAGAAGGTTCGGGAGCTGA; ATGGGTCGTTCACTGCTACC; 113 bp). PCR conditions were as follows: initial denaturation at 95 °C for 2 min. followed by 35 cycles of denaturation at 95 °C for 20 s, annealing at 60 °C for 15 s and extension at 72 °C for 15 s. A final extension step at 72 °C for 5 min was performed and PCR products were then electrophoretically separated in 2% agarose gel in TAE buffer. PCR products of specific sizes noticed on gel confirmed positive expression of all analyzed markers (MKRN1, SPTRX-3 and PAWP) in ram testis (Figure A1C).
